# Supplementary material for: Graph Neural Networks for Carbon Dioxide Adsorption Prediction in Aluminum-Substituted Zeolites
Source: ACS Appl Mater Interfaces. 2024 Oct 2;16(41):56366–75. doi: 10.1021/acsami.4c12198 (PMC11492174; doi:10.1021/acsami.4c12198)
Supplement: Supplementary file 1 — am4c12198_si_001.pdf [file am4c12198_si_001.pdf]

Supporting Information to Graph Neural Networks for Carbon Dioxide Adsorption Prediction in Aluminium-Substituted Zeolites

Marko Petković, José Manuel Vicent-Luna, Vlado Menkovski, and Sofía Calero*

Eindhoven University of Technology, 5612AZ Eindhoven, Netherlands.

E-mail: s.calero@tue.nl

Zeolite Generation

To generate aluminium substituted zeolites, we have used the algorithms introduced in Romero-Marimon et al.[1]. In total, four algorithms for creating aluminium substitutions in all-silica zeolite were introduced. These algorithms place aluminium substitutions using various rules, which leads to different types of Si/Al configurations, which in turn can have varying effects on the CO_2 adsorption within a zeolite. In Figure S1, three examples of generated structures with four aluminium substitutions using each algorithm are presented (for chains, two chains of two were used).

The *random* algorithm chooses (a user specified amount of) random silicon atoms to be replaced with aluminium. As such, the structures generated with this algorithm do not follow a clear pattern.

The remaining algorithms make use of the zeolite graph when generating structures. Here, the nodes represent the T-atoms. Edges are drawn between atoms in cases there is a T-O-T bond between them.

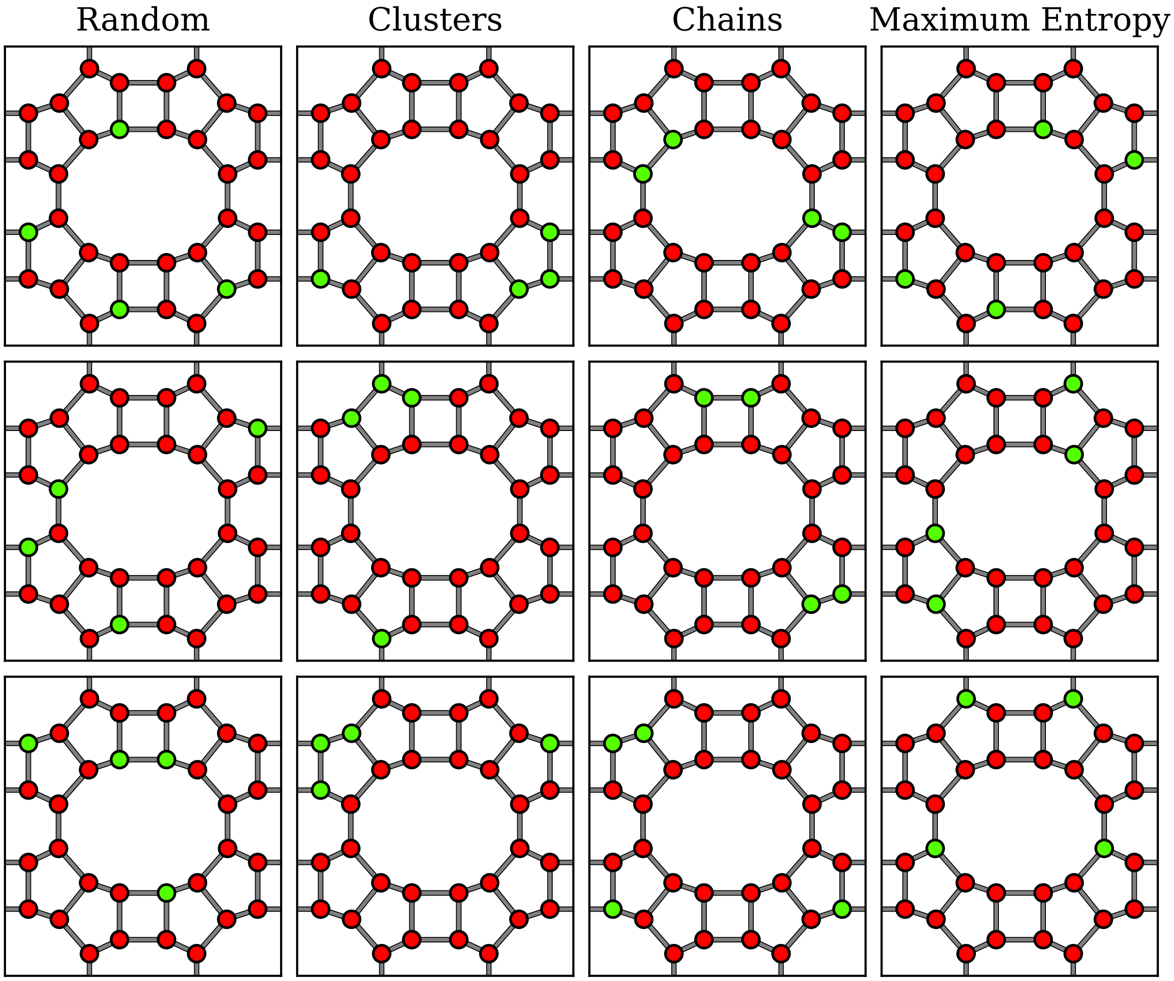

Figure S1: Three examples of MOR structures generated with each algorithm for 4 aluminium substitutions. Silicon atoms are red, while aluminium atoms are green. For simplicity, the structures are visualized in 2D. Note that clusters and chains may go through the periodic boundary.

In the *clusters* algorithm, a random initial atom is selected. Then, the neighbours of the initial atom are also substituted. Neighbours of existing aluminium atoms keep getting substituted, until the amount of user selected aluminium atoms has been reached. In case there are too many atoms in the last set of added neighbours, a random subset is selected to ensure the structure has the user selected number of aluminium atoms. Structures generated by this algorithm contain a high amount of non-Löwenstein bonds.

The *chains* algorithm places the aluminium atoms in chains throughout the zeolite. In addition to the amount of substitutions, the user should also specify the amounts of chains

and their lengths. The algorithm initially places a random atom, and starts constructing the first chain from there. Certain constraints are in place, such as atoms being only allowed to be connected to two other atoms, to prevent the chain from being turned in a "cluster". Once the first chain is finished the algorithm starts with the second chain, by selecting a new random starting atom. Here, it is made sure that the different chains do not connect to each other. This process is repeated until all chains are created.

Finally, the *maximum entropy* algorithm places aluminium substitutions (roughly) uniformly throughout the zeolite. This is done through a stochastic random walk over the graph of the zeolite, where a substitution is placed every set amount of steps. As such, the average one-to-one distance between aluminium atoms is maximized. Full details of the random walk implementation can be found in Romero-Marimon et al.[1].

Parity Plots for Individual Topologies

Parity plots for the heat of adsorption and Henry coefficient for each topology are presented in Figure S2. Here, we see that for all topologies, most predictions by the ML model are accurate. For the lower and higher heat of adsorption and Henry coefficient values, we see that for the MFI and ITW topologies the model sometimes over- or under-estimates the true values. There are several factors which can cause this change in performance, such as the lack of similar patterns in the training dataset, or the complexity of the zeolite. In turn, this causes the higher MSE values in Tables 3 and 4 for MFI and ITW, while the MAE remains lower, since most predictions are still accurate.

Running Time Comparison

To compare the running time of the Monte Carlo (MC) simulations and Machine Learning (ML) model we ran experiments on a AMD Ryzen Threadripper PRO 5975WX CPU (64 thread). To ensure a fair comparison, the inference using ML was done on the CPU instead

Table S1: Average time for property prediction of 60 structures. Numbers between brackets indicate the minimum and maximum running time for MC.

	ML	MC	
MOR	0.12s	9.9h	(2.5h - 16.4h)
MFI	0.68s	8.3h	(2.0h - 15.5h)
RHO	0.09s	3.7h	(1.4h - 5.8h)
ITW	0.05s	6.7h	(1.6h - 12.9h)

of the GPU. Since the RASPA simulations each occupy one thread, it was possible to run 60 simulations in parallel. Therefore, we compare the two algorithms by investigating the time needed to calculate the CO_2 heat of adsorption and Henry coefficient for 60 structures. Since the MC simulations scale with the amount of moving atoms, we also report the minimum and maximum times for MC in Table S1.

Overall, we can see that the ML algorithm is orders of magnitude faster at predicting properties. However, it should be noted, that the ML model first needs to be trained, for which training data from MC simulations is needed. As a result, the amount of structures for which properties are obtained first increases at the same rate for both algorithms. Once the ML model is trained (hours), the rate at which properties of new structures can be predicted will be drastically increased. This process is illustrated in Figure S3, where initially properties for new structures are predicted at the same rate, following which no new structures are predicted by ML during training time. Since both the training and inference using the ML algorithm is fast, the amount of structures for which properties are predicted quickly surpasses the amount of MC.

References

(1) Romero-Marimon, P.; Gutiérrez-Sevillano, J. J.; Calero, S. Adsorption of Carbon Dioxide in Non-Löwenstein Zeolites. *Chemistry of Materials* **2023**, *35*, 5222–5231.

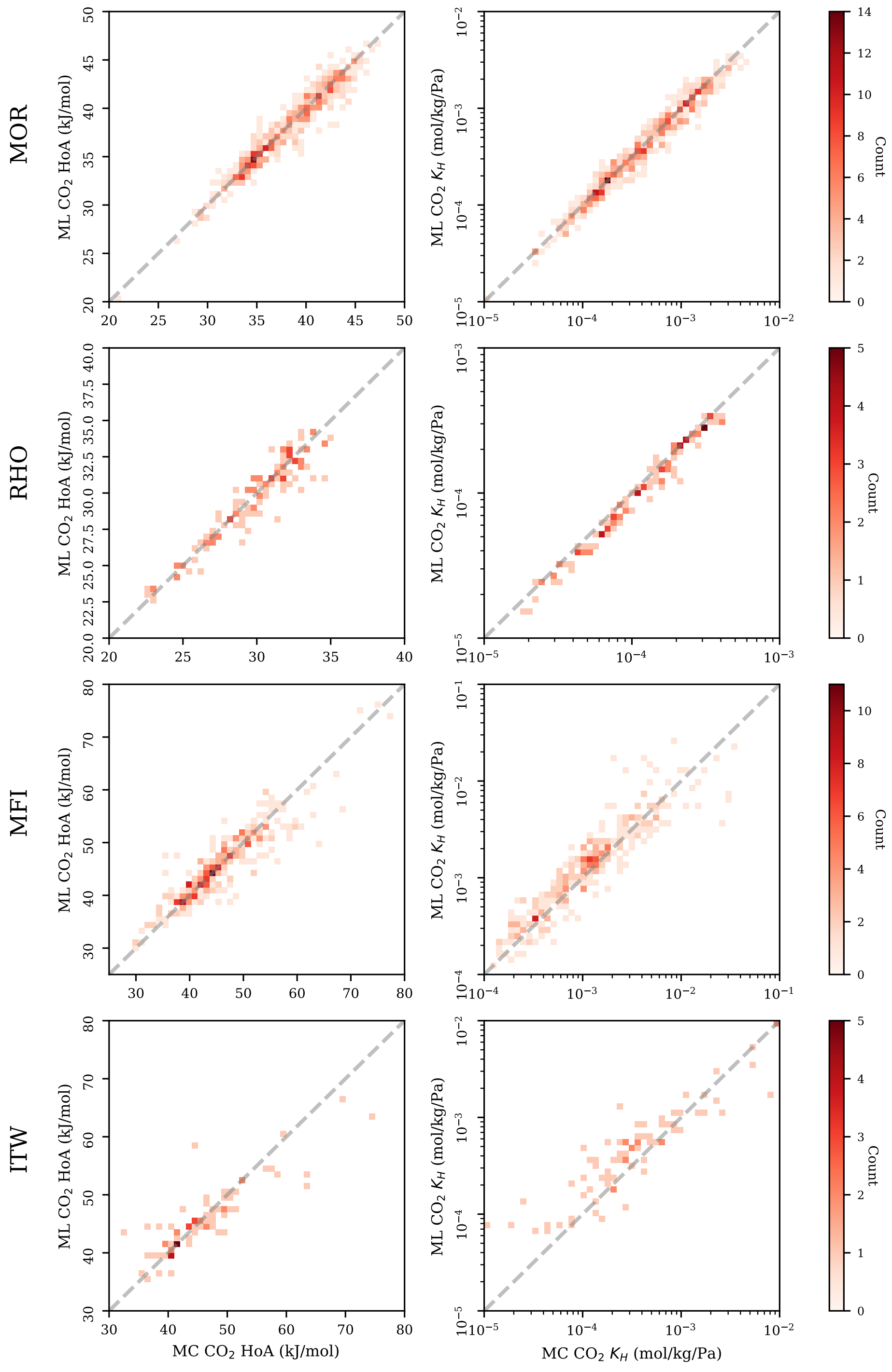

Figure S2: Heat of adsorption and Henry coefficient for individual topologies predicted by MC and ML on the test set.

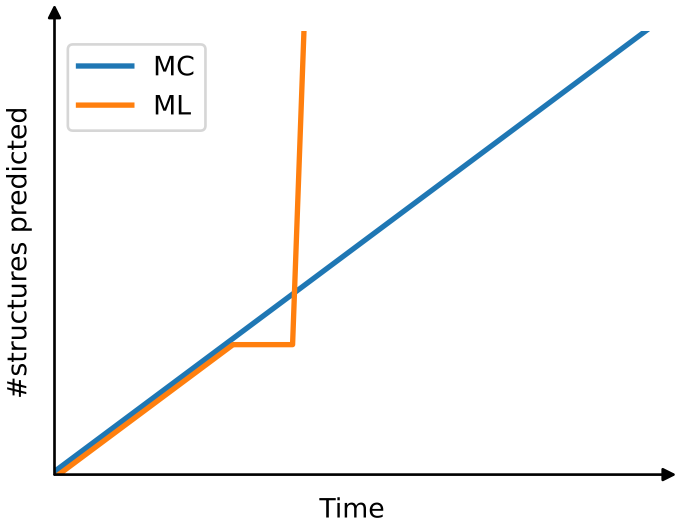

Figure S3: Number of structures for which the properties are predicted over time.
